# In Situ STM Study of Roughening of Au(111) Single-Crystal Electrode in Sulfuric Acid Solution during Oxidation–Reduction Cycles

**DOI:** 10.1021/acs.jpcc.4c06362

**Published:** 2024-10-17

**Authors:** Saeid Behjati, Marc T. M. Koper

**Affiliations:** Leiden Institute of Chemistry, Leiden University, PO Box 9502, 2300 RA Leiden, The Netherlands

## Abstract

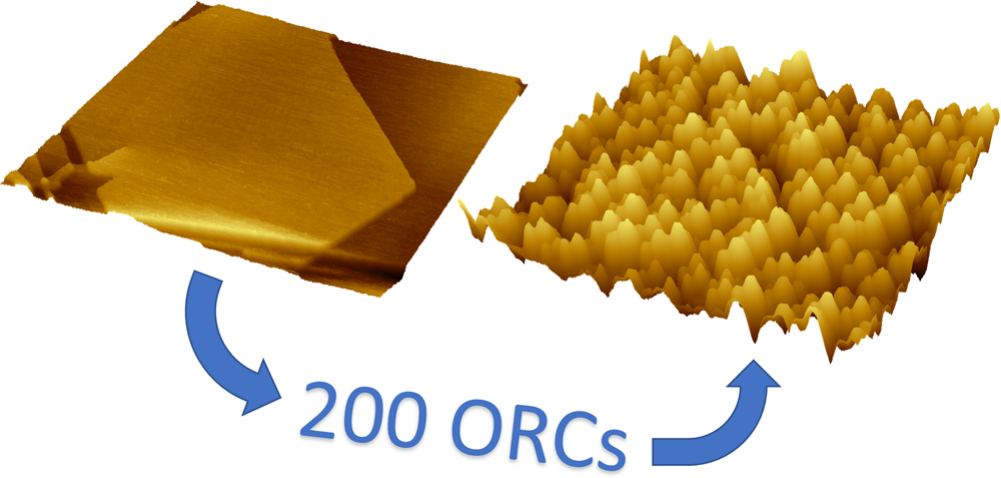

Oxidation–reduction cycles (ORCs) on Au(111) in 0.1 M sulfuric acid solution change the electrode morphology due to the formation of many new nanosized islands. With increasing the cycle number, the roughness of the surface increases due to the formation of multiatomic-step adatom islands and pits. The final roughness value is a function of the applied potential window, number of ORCs, scan rate, electrolyte concentration, and any applied delay time. In a first experiment, the roughening was tracked by recording the STM images in 11 steps during 200 ORCs. The results show the formation of pyramidal islands and a linear correlation between the roughness amplitude and the cycle number. In a second experiment, the 200 cycles were studied in 38 steps, while after each step, two images were recorded with a 3 min delay by holding the potential in the double-layer window. This leads to a lower roughness increase due to the high surface mobility of the Au surface atoms, which smoothens the surface during the delay time. Finally, the oxidation–reduction charge density per cycle shows an inverse correlation with surface roughness due to the (111) terrace showing a higher surface oxidation charge than the other sites and facets. Each delay causes a strong increase in the oxidation charge which is a consequence of surface smoothening during the delays leading to an enhancement of the (111) related oxidation charge.

## Introduction

Gold is well-known as an important material for various applications, and therefore the structure and characteristics of single-crystal gold surfaces have been studied in ultrahigh vacuum (UHV)^1,2^ and aqueous electrochemical environments.^3,4^ Electrochemical characterization of gold electrodes often involves cyclic voltammetry experiments in which the gold surface is oxidized and subsequently reduced. Oxidation of gold electrodes in acidic electrolytes containing sulfate or perchlorate anions has been studied thoroughly.^4−7^ For sufficiently positive potential, sulfate anions will be specifically absorbed on the surface and form an ordered sulfate adlayer, with the anions undergoing either partial or complete charge transfer.^8^ On the other hand, perchlorate anions are considered to reside in the electrochemical double layer without chemisorption and adlayer formation. The onset potential for surface oxidation is influenced by this adlayer, as anion adsorption blocks the initial stages of OH electrosorption. At sufficiently positive potential, two reactions have been suggested to initiate surface oxidation:
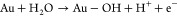
1
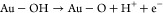
2As shown in Reaction 1, the oxidation starts with electroabsorption of OH^–^. Reaction 2 will take place at slightly more positive potential and has been suggested to lead to a two-atom thick oxide layer.^6^ The formation of Au–OH, Au–O, and Au_2_O_3_ species on the surface has been considered based on charge and capacitance results acquired by conventional electrochemical techniques.^5,9,10^

By applying successive oxidation–reduction cycles (ORC) on Au (111) single crystal electrodes in an acidic electrolyte, some long-range nanopatterned surfaces are formed which after many cycles will lead to a highly roughened surface. This procedure is used e.g., for preparing rough gold surfaces for Surface Enhanced Raman Spectroscopy.^11^ In situ electrochemical scanning tunneling microscopy (EC-STM) is a suitable technique to record the surface evolution with atomic resolution from the pristine Au (111) surface to the final roughened or electrochemically annealed surface.^12^ The pattern formation resulting from the applied oxidation–reduction cycles likely depends on many parameters; specifically: the lower and upper potential limits of the cycles, the potential scan rate, the number of cycles, and the electrolyte composition. It has been reported that after 10 ORCs in 0.1 M sulfuric acid, only vacancy islands form on Au(111), and this points out that some other mechanisms are taking place (i.e., gold dissolution) apart from the place exchange mechanism. By increasing the number of cycles, adatom islands start to appear.^3^ In this paper, we perform a detailed study and analysis of the oxidation–reduction cycling experiment to have a better understanding of the roughening process. Specifically, we study how stopping the potential in the double layer window during the cycling strongly affects the gold surface dynamics, showing that the gold surface atoms are highly mobile even if surface oxidation and reduction do not take place. This has a corresponding effect on the roughening of the surface. Moreover, we show that the roughening stages are not well captured quantitatively by the oxide formation and reduction charges from the cyclic voltammetry.

## Experimental Section

### EC-STM Measurements

The Electrochemical Scanning Tunneling Microscope (EC-STM) images were recorded with a home-built instrument, which was developed at Leiden Institute of Chemistry (LIC) of Leiden University. Details of the design and construction of this instrument are given in the Supporting Information. The tips were produced from a platinum/iridium wire (90/10) by the pulling-cutting method. To reduce the extra faradaic current on the tip, a layer of hot melt adhesive (EVA-copolymer, synthetic resin, Wax and Stabilizer Brand: C.K.) was added except for the apex of the tip. A disk-shaped single-crystal electrode Au(111) (10 mm diameter) with a gold wire welded at the back was used as the working electrode (WE). The crystal was cut with an accuracy of 0.1° and polished down to 30 nm roughness (Surface Preparation Laboratory, Netherlands). Before each measurement, the Au(111) sample was annealed by a butane flame torch to an orange color for 5 min and cooled down in air above the surface of ultrapure water to avoid introducing contaminations to the sample surface. A high-purity gold wire was used as the counter electrode (CE) and a reversible hydrogen electrode (RHE, Hydroflex, Gaskatel) was used as a reference electrode (RE). The distance between the working electrode and the reference electrode is about 7 mm to minimize the ohmic drop during the voltage sweep. The images were recorded in constant tunneling current mode with the tunneling bias between 10 and 20 mV and the current set point was between 100 and 150 pA. The current set point was changed to zero to maximize the distance between the tip and the sample while applying the cyclic voltammetry (CV). Then the electrochemical voltage was set to the “rest potential”. In this condition, by increasing the current set point, the tip could approach, and the tunneling current on the tip appeared. During the experiment, the EC-STM chamber was purged with ultrahigh-purity argon gas to reduce the chance of oxygen (or other gases) dissolving into the EC-STM cell.

### Electrochemical Cell and Electrolyte

A custom-made Pyrex glass cell was used for standard electrochemical experiments. The glassware and plastic parts were cleaned by leaving them in a permanganate solution (0.5 M sulfuric acid and 1 g/L potassium permanganate) for at least 12 h prior to each experiment. After rinsing them with milli-Q water, diluted piranha solution (3:1 mixture of sulfuric acid (H_2_SO_4_) and hydrogen peroxide (H_2_O_2_), diluted with water) was used to remove the manganese oxide and permanganate. By boiling all the parts at least five times, the residue of diluted piranha was removed. The electrolyte contains H_2_SO_4_ (96%) Suprapure Sigma-Aldrich) and was prepared by usage of ultrahigh purity (UHP) milli-Q water (resistivity > 18.2 MΩ cm). It was degassed with ultrahigh-pure argon gas for at least 30 min. All the measurements took place at room temperature (*T* = 293 K).

## Results and Discussion

### Oxidation–Reduction Cycles of Au(111) without Holding Potential in Double Layer

**Figure 1:**
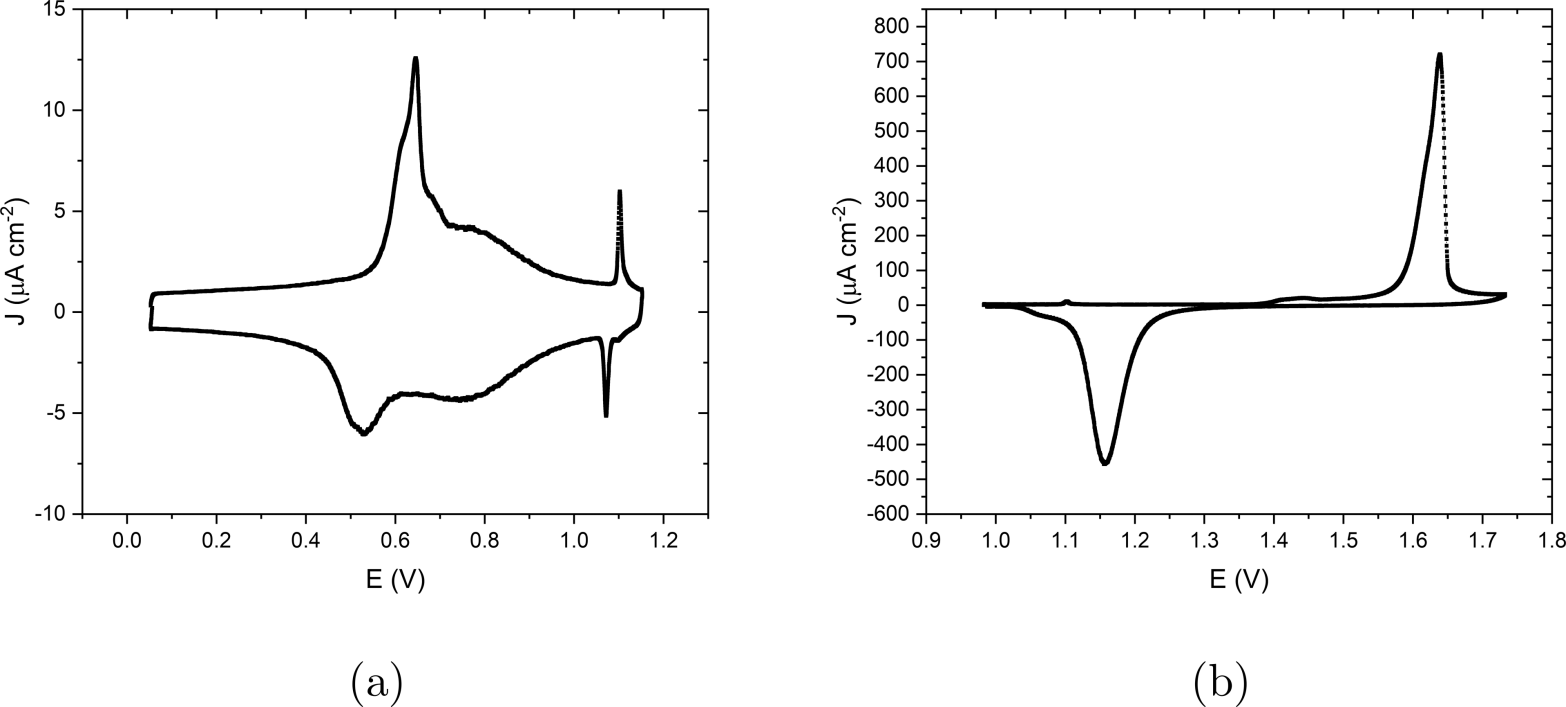
Cyclic voltammetry for Au(111) electrode in 0.1 M sulfuric acid with a scan rate of 50 mV s^–1^ at a temperature of *T* = 298 K (a) from 0 to 1.15 V versus RHE and (b) from 0.98 to 1.7 V versus RHE.

To check the quality of the sample surface, Figure 1a shows the CV of the Au(111) electrode in 0.1 M sulfuric acid that was recorded with a scan rate of *s* = 50 mV/s in the potential window of 0–1.15 V. At potentials below 0.5 V, there is a low current corresponding to the double layer charging of the () reconstructed surface, which was formed during the annealing step. At higher potential (ca. 0.55 V), sulfate adsorbs and induces the lifting of the reconstruction, corresponding to the anodic peak at 0.64 V.^7,13^ The broad peak which appears at 0.78 V is due to further absorption of anions and lifting the rest of the reconstruction.^14^ The subsequent sharp anodic peak at 1.10 V is caused by the formation of an absorbed anion overlayer with () R19.1° structure.^7^ In the reverse scan, the peaks at 1.07, 0.68, and 0.53 V correspond to the reverse processes, with the reformation of the reconstructed surface at the lowest potentials. By increasing the upper potential of the voltage window, the CV in Figure 1b was recorded. There is now one distinct anodic peak at 1.62 V, corresponding to surface oxide formation and anion desorption. With the presence of defects on the surface, a small and broad peak at 1.44 V appears and its absence would indicate the quality of the sample used in the experiment.^3^ Finally, the large cathodic peak at 1.16 V in the return scan shows the reduction of the formed oxide and the reabsorption of anions. The shoulder peak observed at potentials negative of the main cathodic peak has been discussed as resulting from the formation of two different oxides.^15^

Next, a series of experiments was conducted to investigate the roughening process occurring during oxidation–reduction cycles. The same sulfuric acid electrolyte was used in the EC-STM experiments. Duplicate experiments were performed to confirm the reproducibility of the experimental observations.

**Figure 2:**
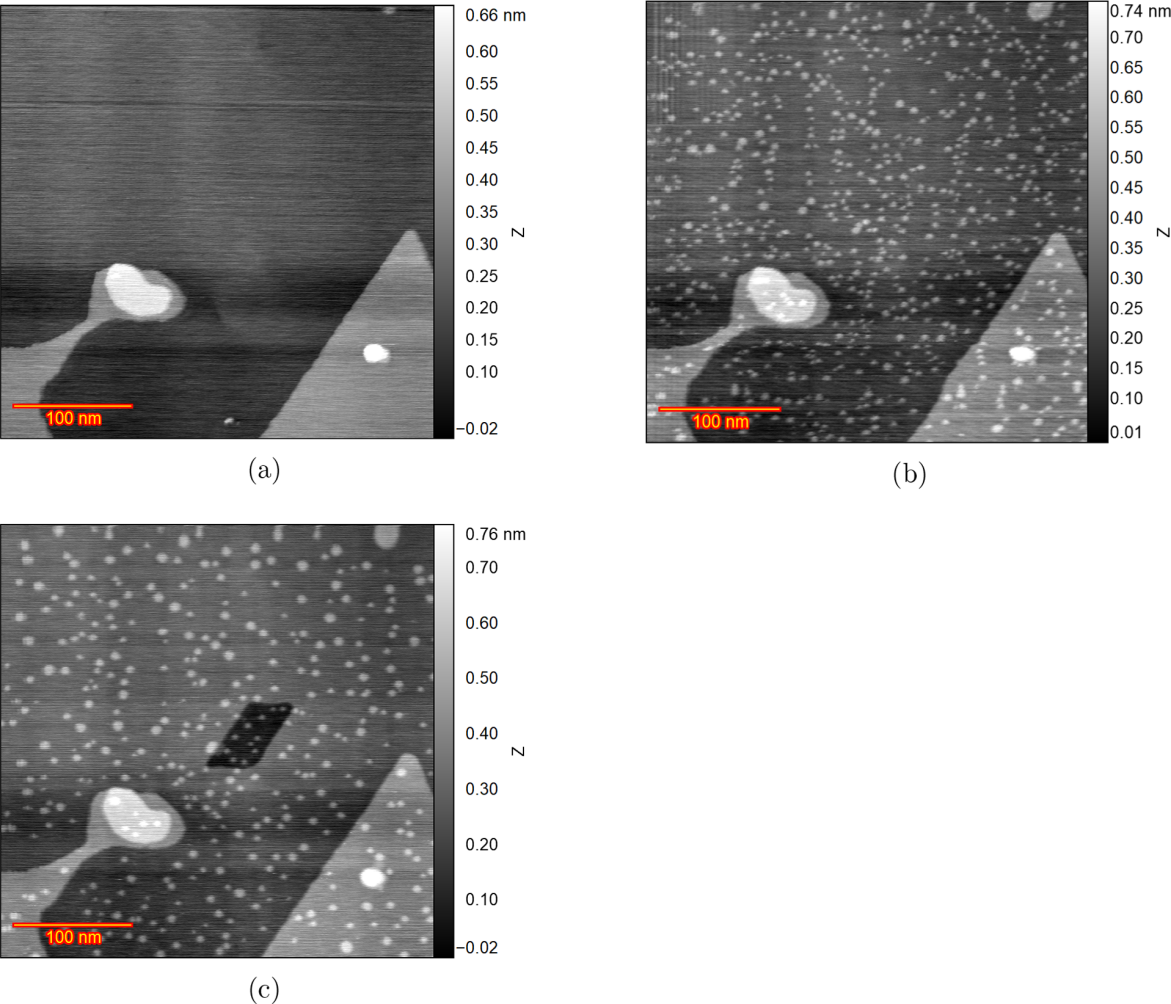
EC-STM images of Au(111) in 0.1 M Sulfuric acid with the image size of 350 × 350 nm for the experiment without holding potential in double layer (a) Pristine surface after being thermally reconstructed at 0.05 V. (b) Lifting the reconstruction by applying a higher potential of 0.88 V leading to the formation of small islands. (c) Recorded image after a 4 min delay after (b) without changing any parameters.

Figure 2a shows an EC-STM image of the pristine surface of the sample in 0.1 M sulfuric acid at an electrode potential of 0.05 V. There are some defects in the left-bottom and right-bottom parts of the image which are useful to aid in the compensation for the thermal drifts in long-term experiments. There are still large flat terraces in the middle and top parts of the image, which is a good situation for studying the roughening of the Au(111) surface. Figure 2b shows the Au(111) surface after the potential has been swept from 0.05 to 0.88 V. As the potential is higher than the potential of zero charge, the reconstruction of the Au(111) surface has been lifted. The lifting of the reconstruction causes ca. 4% of excess atoms in the first atomic layer to be expelled, resulting in the formation of numerous small islands. The formed islands are monatomic (ca. 2.3 Å). Figure 2c is recorded at the same potential of 0.88 V after a 4 min delay, to show the influence of time for comparison with Figure 2b. The number of islands has evidently diminished, while simultaneously observing an increase in their size, suggesting the ripening of the islands. The rapid growth of the islands shows that gold atoms are rather mobile and can detach from the steps and hoover on the terraces. They finally find each other, or other islands, and form larger islands. Additionally, a large vacancy island (parallelogram shape) has appeared on the initially flat terrace. The reason for the appearance of this large vacancy island is unknown, but it could be caused by some crystal defects in bulk.

**Figure 3:**
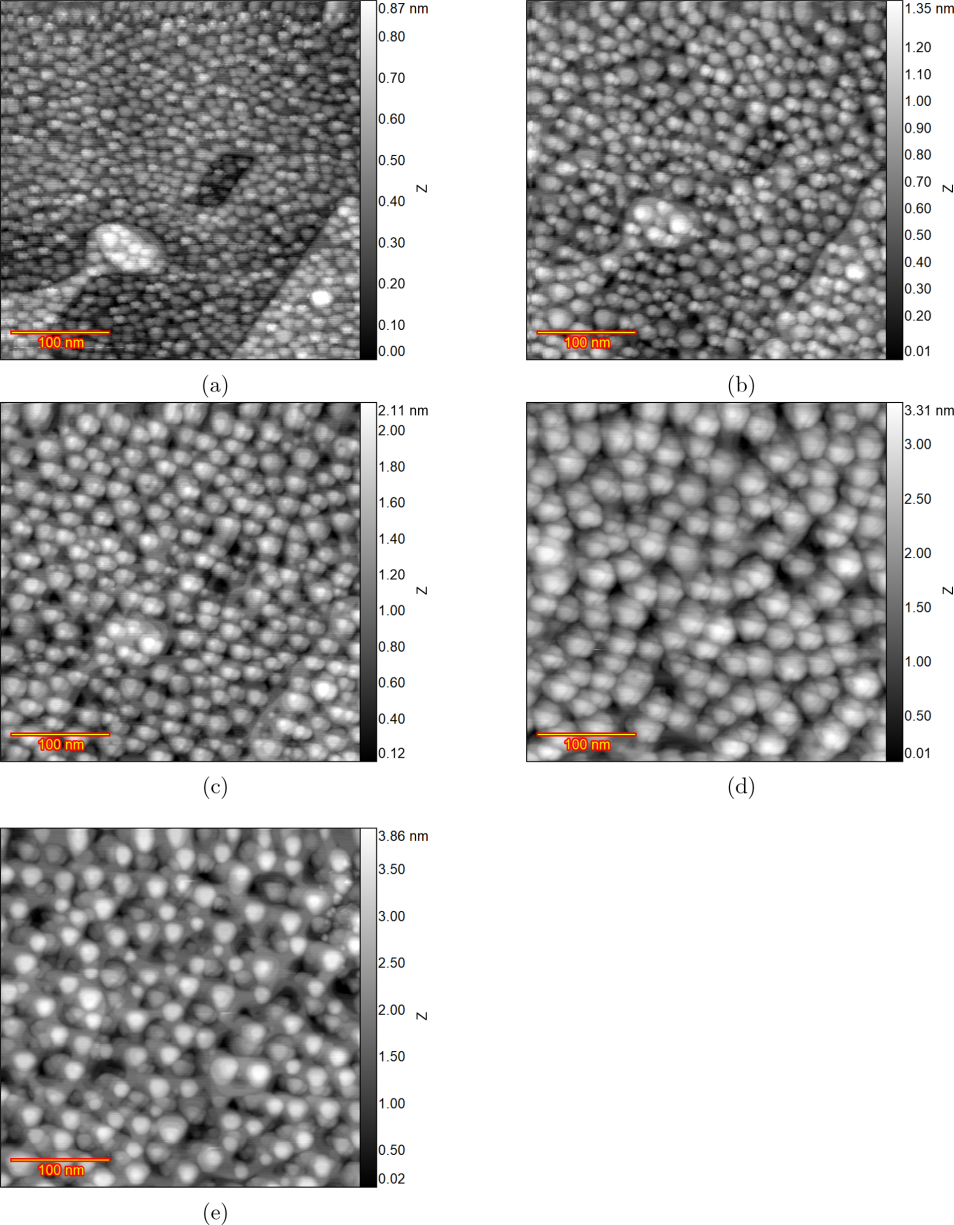
EC-STM images of Au(111) in 0.1 M sulfuric acid with the image size of 350 × 350 nm for the experiment without holding potential in double layer after, (a) 5, (b) 10, (c) 50, (d) 150, and (e) 200 cycles from 0.98 to 1.73 V with a scan rate of 50 mV s^–1^. All the mages were recorded at 0.98 V and each image collection takes c.a. 2 min.

Figure 3a shows the sample surface after 5 consecutive oxidation–reduction cycles from 0.98 to 1.73 V with a scan rate of 50 mV s^–1^ with the image recorded at 0.98 V. The formation of numerous islands with varying heights of one or even two atomic steps is evident throughout the surface in Figure 3a. Additionally, the presence of vacancy islands between these islands can be observed. After five additional consecutive cycles (i.e., total number of cycles reaching 10), the image in Figure 3b was recorded. Comparing the images after 5 and 10 ORCs, a distinct and noteworthy trend emerges: the number of islands with two atomic steps in height has visibly increased, while the presence of monatomic islands appears to be scarce after 10 cycles. The roughening process has progressed to the extent that one can barely distinguish the original surface at this stage, though interestingly enough, the parallelogram can still be distinguished. Figure 3c shows the STM image after 50 cycles when the islands have grown in size while having diminished in number. The island shape is approximately triangular and their direction is not randomly distributed. After 150 ORCs, Figure 3d is recorded and shows that features like the step lines of the initial surface are not distinguishable anymore, and the entire surface is covered by larger pyramids oriented to the three crystallographic axes (particularly [110], [101], and [011] directions). Moreover, their height is increasing indicating an increase in surface roughness. The surface after 200 ORCs is shown in Figure 3e. At higher ORC numbers, it is evident that the height of the pyramids is increasing (considering the gray scale bar range). For a more quantitative comparison, it is necessary to calculate the average size and height of the islands for each stage. An appropriate approach for this is to calculate the height–height correlation function.

### Calculation of the Height–Height Correlation

**Figure 4:**
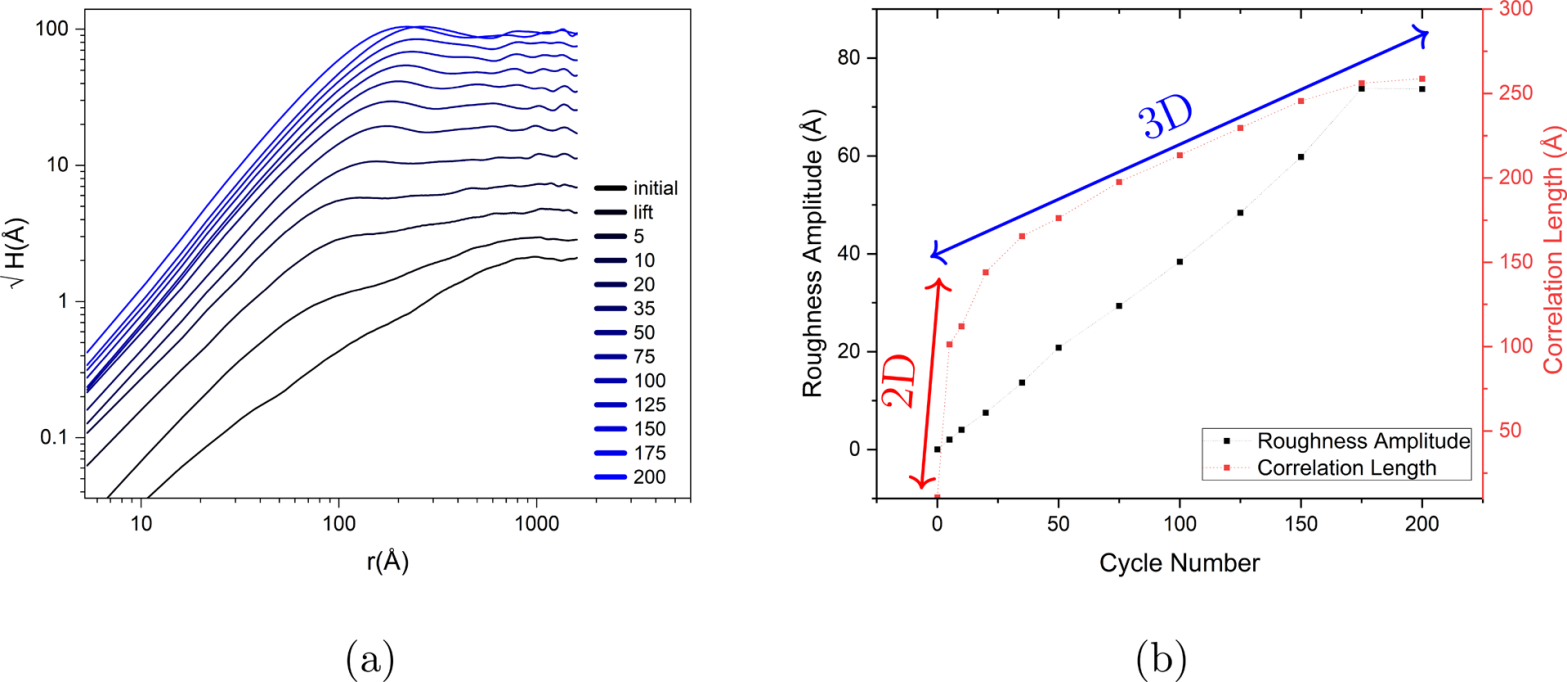
(a) Height-height correlation function versus distance r for Au(111) in 0.1 M sulfuric acid as a function of the number of oxidation–reduction cycles (ORCs) for the experiment without holding potential in the double layer. (b) Extracted roughness amplitude and correlation length versus cycle number from the HHCF results. The arrows indicate the 2D and 3D island growth regimes.

Determining the height–height correlation function (HHCF) serves as a robust analytical tool for characterizing the spatial properties and scaling behavior of surface roughness. Height-height correlation relies on the principle that the heights of neighboring points on the surface are not independent but correlated. This method quantifies the level of correlation or similarity between the heights of distinct points in relation to their separation distance. The formula for the calculation of the HHCF is
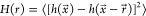
3
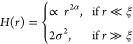
4where *h*(*x⃗*) is the surface height at the given position *x⃗* and *h*(*x⃗* – *r⃗*) is the height for a position at a distance of *r⃗* from position *x⃗*. For a self-affine surface, the HHCF has the form of eq 4.^16^ From this equation, the root-mean-square (rms) roughness amplitude (σ), the lateral correlation length (ξ), and the roughness exponent (α) can be derived through the analysis of the HHCF.^17^ The size of the analyzed images must be at least 10 times greater than the lateral correlation length (ξ) to ensure statistical relevance in the HHCF calculations. In order to facilitate a meaningful comparison among all frames, a linear regression was employed on the images to mitigate sample tilt effects (the tip is not perfectly perpendicular to the sample surface). This prevents the sample tilt from introducing extraneous roughness into our analysis. The height–height correction function thus calculated is depicted in Figure 4a. For large *r⃗*, a plateau emerges, providing the basis for calculating the root-mean-square (rms) roughness amplitude (σ) (see eq 4). With an increase in the number of oxidation–reduction cycles (ORCs), the roughness increases, in agreement with the topographical images. For smaller *r⃗*, a distinct slope is observed, from which the roughness exponent (α) can be calculated. The distance at which the transition occurs from the slope to the plateau can be regarded as the lateral correlation length, (ξ). First of all, at a low number of ORCs, it is observed that *h*(*x⃗*) shows (at least) two slopes. In the case of the first frame (representing the pristine surface), we expect the lateral correlation length to be very large (ideally infinite). However, in practice, there are steps, defects, and electrical and mechanical noises in the image. Therefore, the pristine sample surface shows a correlation length of ca. 100 nm. Once the reconstruction is lifted, we expect to have some islands with specific diameters on these terraces. There is an intermediate range of ORCs in which the transition from the sloped line to the plateau takes place in two steps. This is a result of having two scaling regimes.^18^ There is a steeper part at small *r⃗* which is due to the roughening caused by the ORCs (microtexture), followed by an intermediate region with a different slope that is related to the steps and the defects in the frame (macrotexture). Under these conditions, we assign the correlation length of the new monatomic islands as the transition point from the steeper line to the less steep line. The second transition point matches the correlation length of ca. 100 nm of the pristine surface. To avoid having two scaling regimes, one could ideally avoid having any steps or defects in the analyzed area. After more ORCs, the surface becomes more roughened and consequently, the roughness of the initial steps and defects of the surface becomes insignificant. Figure 4b depicts the roughness amplitude (in black) and correlation length (in red) versus the cycle number. The roughness amplitude (σ) increases linearly with the cycle number (except the 200th cycle). For the correlation length (ξ), one can divide the curve into two regimes with different slopes. This shows that in the initial stage of roughening, the islands grow faster laterally (corresponding to the correlation length) and as soon as they reach the size of ca.15 nm, their lateral size changes more slowly and they tend to grow mainly in height.

### Oxidation–Reduction Cycles of Au(111) with Holding the Potential in Double Layer

In the next experiment, we aimed to investigate the effect of a waiting time in the double layer region on the roughening induced by ORCs of the Au(111) single crystal in 0.1 M sulfuric acid. The goal of this experiment is to study the effect of gold atom surface dynamics in the double-layer window on a roughening surface. Before this particular experiment, we also studied the potential-induced lifting of the reconstruction in more potential steps. This is described in the Supporting Information. Figure S1 shows the strip reconstruction (which forms at higher strain values^19^), early adatom island formation, step line evolving, formation of monatomic islands after lifting, and island ripening.

**Figure 5:**
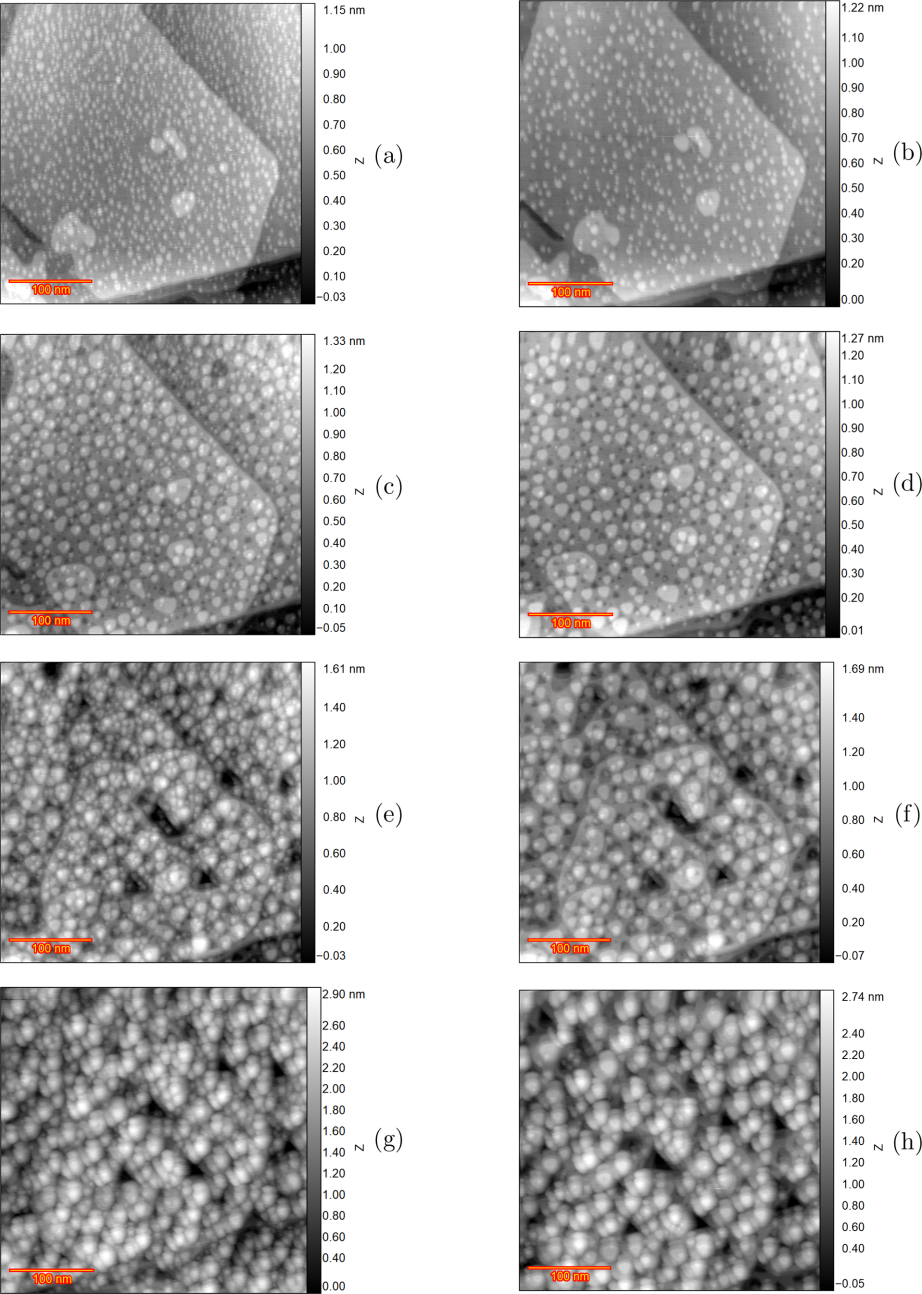
Au(111) in 0.1 M sulfuric acid with the image size of 350 × 350 nm with holding potential in double layer after (a) *N* = 1 instant, (b) *N* = 1 delayed, (c) *N* = 10 instant, (d) *N* = 10 delayed, (e) *N* = 50 instant, (f) *N* = 50 delayed, (g) *N* = 150 instant, (h) *N* = 150 delayed, where N is number of ORCs.

The experiment including delay was as follows. The electrochemical potential was kept at 0.98 V as the lowest vertex potential for the entire experiment. Next, a certain number of oxidation–reduction cycles from 0.98 to 1.73 (V) with a scan rate of 50 mV/s were performed with the tip retracted. Immediately after the number of OR cycles, the tip was approached and an image was recorded. We call this “instant frame”. Image recording takes about 2 min, after which a 1 min waiting time at 0.98 V was applied. Then, another image was recorded which is named “delayed frame”. Therefore, the total time difference for each corresponding scan line in the instant and delayed frames is 3 min. During the waiting time, no parameter was changed to rule out any other sources for disturbance/change of the system with the tip standing in the top-left part of the scan area, in tunneling mode. Figure 5a shows the instant frame after the first ORC. Many small adatom islands and some vacancy islands were formed. The delayed frame is depicted in Figure 5b, which shows the presence of larger islands. The extra atoms for the formation of larger islands come from smaller islands as the result of either Ostwald ripening or Smoluchowski ripening. After the 10th ORCs, the instant frame and the delayed frame in Figure 5c,d were recorded. The total number of the adatom islands increased and many small vacancy islands emerged. Moreover, some bilayer adatom islands are formed. This is the starting point of the 3D island growth, but the delay is postponing that. The delayed frame shows that the islands have increased in lateral size at the expense of smaller islands. Additionally, many small islands in the second layer have also vanished. It seems the decay rate of the top layer islands depends on the size of the bottom layer island underneath. It is known that for multilayer islands on Cu(111), the Ehrlich-Schwoebel barrier remains constant for a terrace width higher than a critical width, but vanishes for the terrace widths lower than that.^20,21^ Similar behavior has been observed on Ag(111).^22^ This can explain why having a bilayer adatom island with a small size is not common and many bilayer adatom islands have larger sizes on the first layer. Moreover, the islands tend to show a triangular shape at this potential with more delay time, they form larger islands that show the equilibrium shape more evidently(see Figure 5d).

Figure 5e shows the surface after 50 ORCs. Apart from the formation of new adatom islands, some large vacancy islands formed mainly at the spots where we observed the formation of early adatom islands during lifting the reconstruction (see in SI Figure S1). Also, a long step line was formed, initiated from the dislocation in the left bottom corner. Figure 5f shows the delayed frame which has fewer small islands. At this stage, multilayer adatom islands are formed. Evidently, the surface becomes rougher by applying more cycles, i.e., after 150 ORCs (Figure 5g), with a delay time always leading to smoothening of the surface (Figure 5h). Even at high cycle numbers, there is a difference between the delayed frame and the instant frame especially where the smaller islands are located, but the changes are more conspicuous at lower cycle numbers.

**Figure 6:**
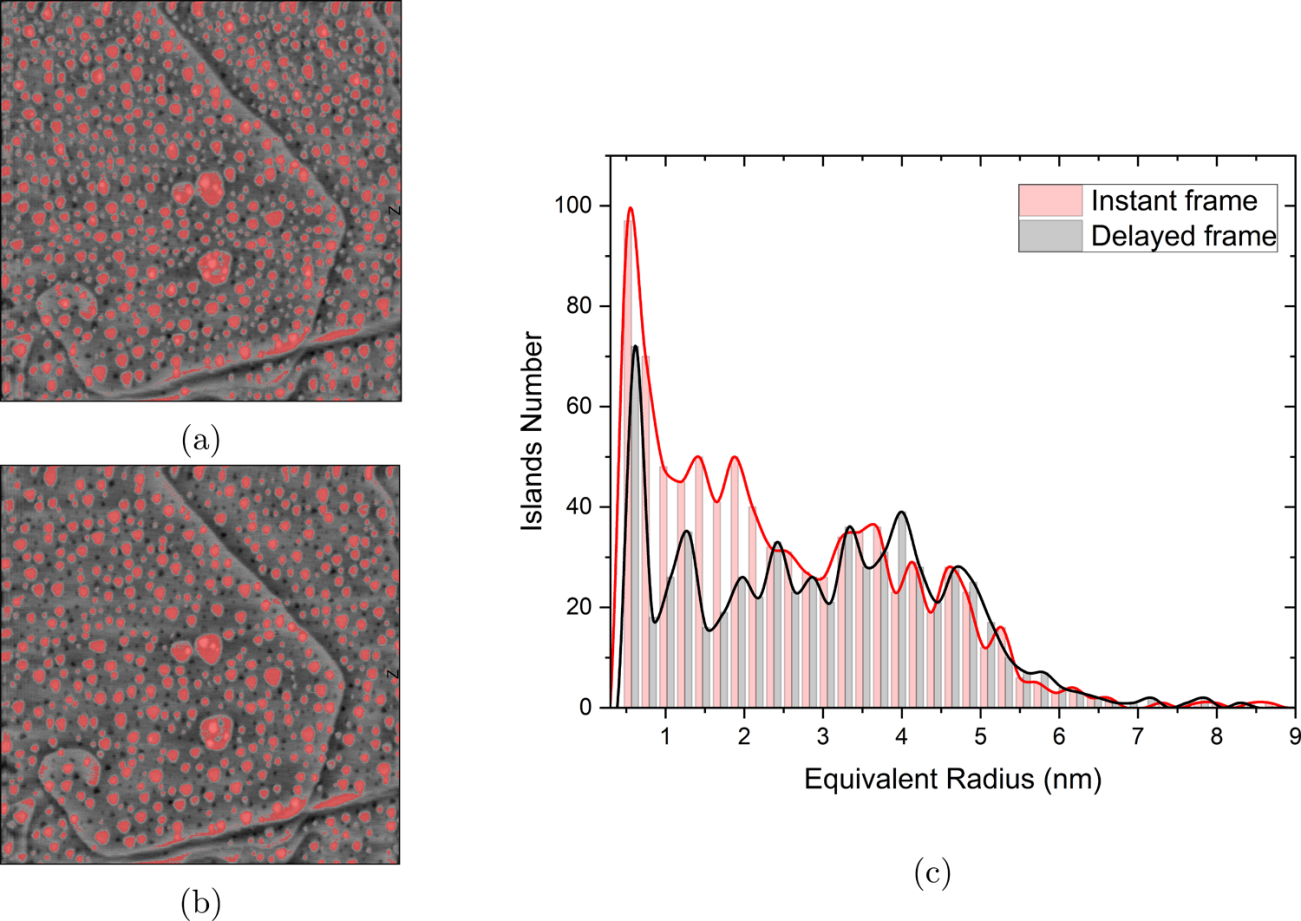
Adatom island detection for the frames after four ORCs with holding potential in double layer (a) instant frame which shows more small islands. (b) Delayed frame. (c) Islands number versus equivalent radius for the instant frame and delayed frame after four cycles. Solid lines connect the end points of the bars for easier comparison.

To have a more quantitative study of this delay effect, detection of the islands and subsequent calculation of their equivalent radius has been performed with Gwyddion software.^23^Figure 6a shows the results of the detected islands (in red) for the fourth ORC instant frame and Figure 6b shows the same results for the delayed frame. From these images, the surface area of the islands was calculated and the equivalent radius of the islands was extracted. Figure 6c depicts the number of islands for the instant frame in red and the delayed frame in black versus their equivalent radius. The bars represent the number of the islands for the corresponding equivalent radius and the solid lines are drawn to facilitate the comparison. From this comparison, it is evident that the delay is specifically reducing the small islands with a radius of less than 2.5 nm, whereas larger islands seem less affected.

### Calculation of the Height–Height Correlation

**Figure 7:**
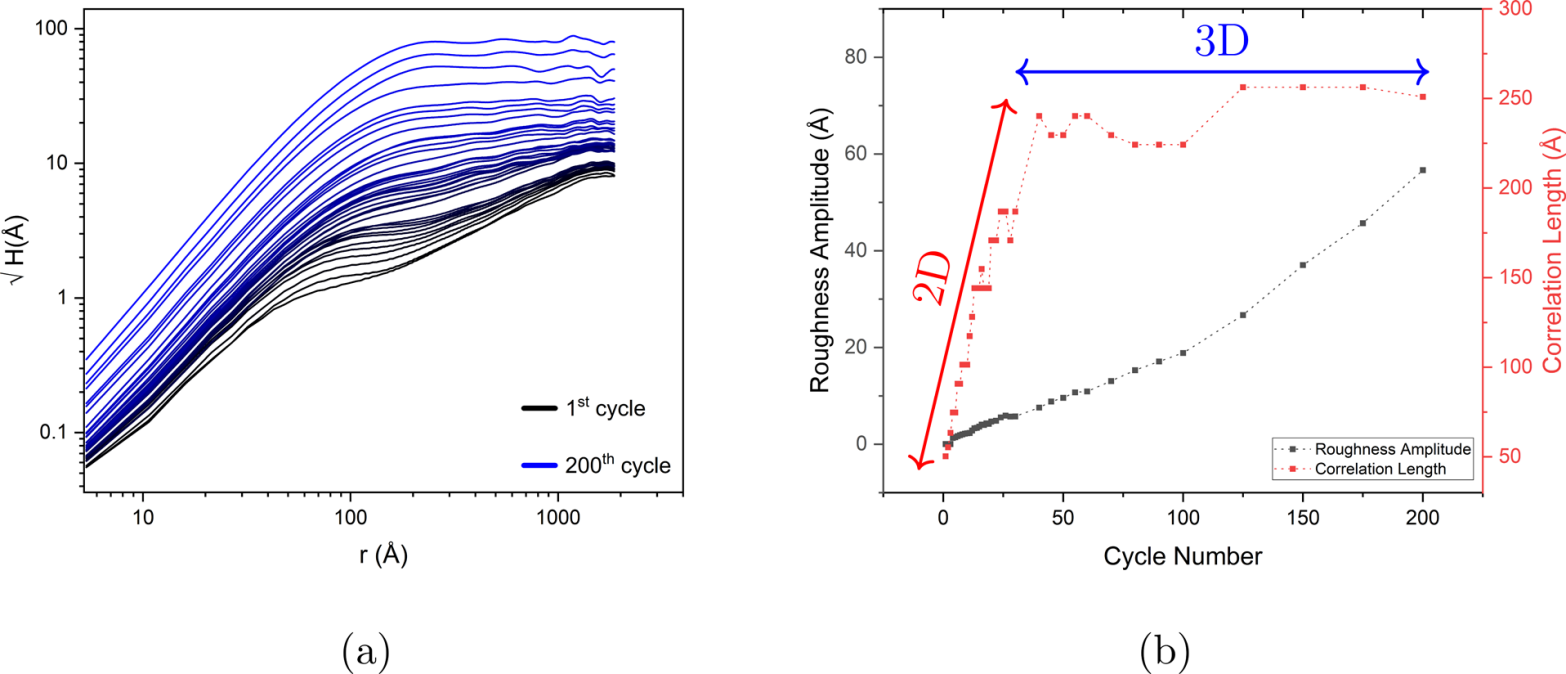
(a) Height-height correlation function versus distance r for Au(111) in 0.1 M sulfuric acid as a function of the number of oxidation–reduction cycles (ORCs) for the experiment with holding potential in the double layer for the delayed frames. (b) Extracted roughness amplitude and correlation length versus cycle number from the HHCF results. The arrows indicate the 2D and 3D island growth regimes.

Figure 7a shows the HHCF for the delayed frames and the color gradient goes from black for the first cycle to blue for the 200th cycle. Figure 7b is the extracted correlation length and roughness amplitude versus cycle number from the HHCF results. The correlation length more clearly indicates the transition between 2D and 3D growth regimes at higher values. The roughness amplitude is increasing linearly with cycle number but the slope is slightly different from the 100th cycle onward because only four delays are applied between 100th cycle and 200th cycle. This change in the slope can be explained easily by comparison of the experiment with and without holding the potential.

**Figure 8:**
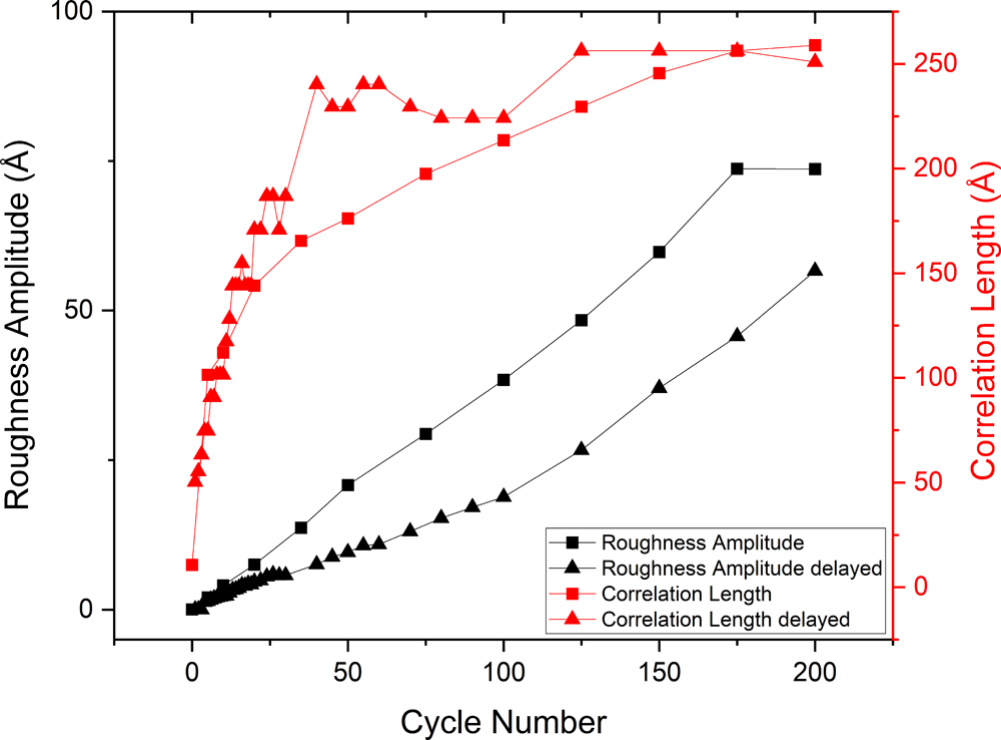
Roughness amplitude and correlation length versus cycle number for the experiment without holding potential in double layer (lines with square data points) and the delayed frames of the experiment with holding potential in double layer (lines with triangular data points).

Figure 8 compares the roughness amplitude and the correlation length versus cycle numbers for the delayed frames of the experiment with holding the potential shown in Figure 7b and for the experiment without holding the potential shown in Figure 4b. The comparison shows clearly the influence of the delay on the development of the roughness amplitude and the correlation length during ORCs. The experiment with additional delay shows a lower slope for roughness amplitude versus cycle number (the black line with triangular data points) and reaches a larger correlation length (ca. 20 nm) at a lower cycle number (the red line with triangular data points). By considering the fact that even for the experiment without holding the potential, there were some inevitable delays because of image recording, it is clear that the eventual roughening of the gold surface is very sensitive to scan rate and the existence of any delay times in the experiments (for instance by recording an image).

### Comparison of STM-Derived Roughening to Oxidation–Reduction Charge Density

**Figure 9:**
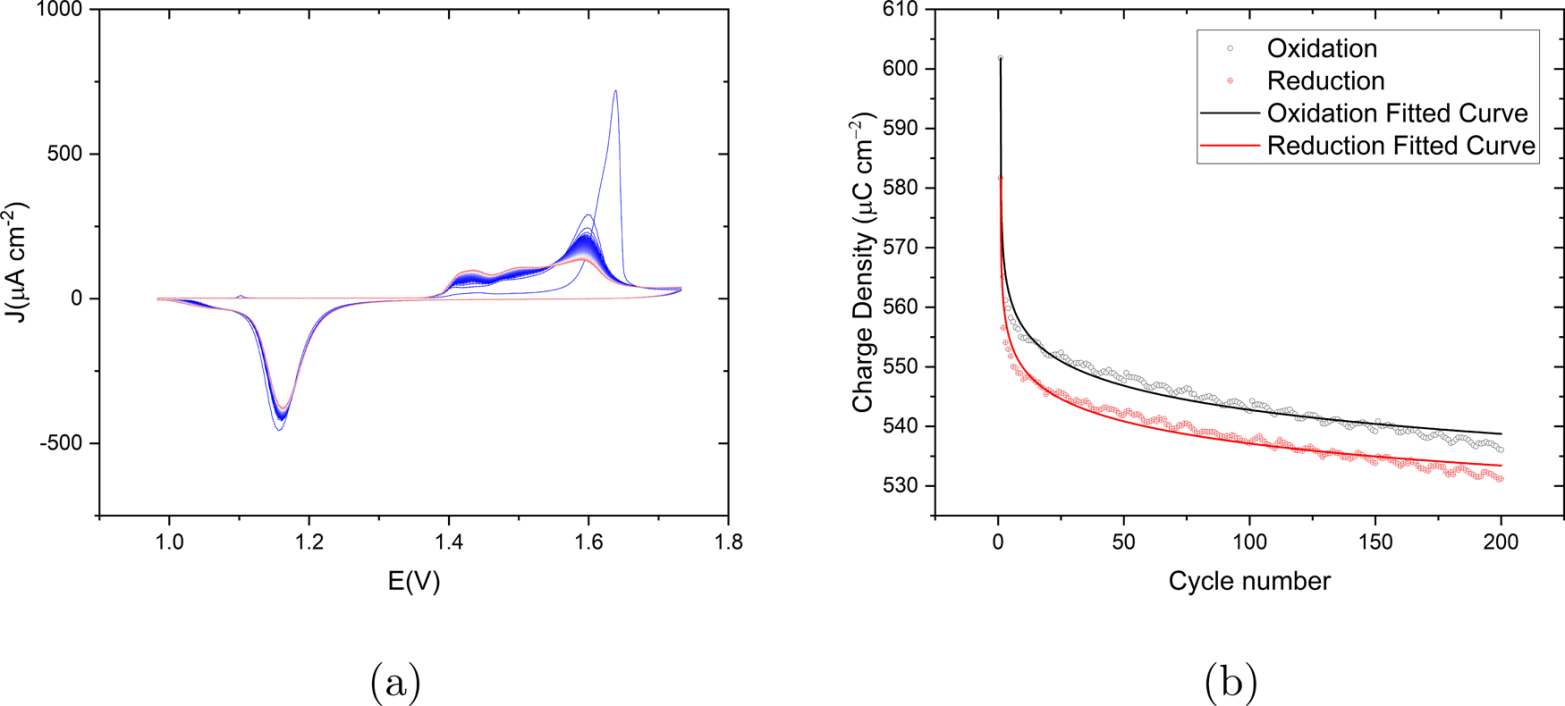
(a) Cyclic voltammograms of the consecutively applied 200 ORCs on Au(111) in 0.1 M sulfuric acid with a scan rate of 50 mV s^–1^ versus RHE. The color gradient from blue to red corresponds to the progression from the first to the last cycle. (b) Circles show calculated oxidation (black) and reduction (red) charge density (μC cm^–2^) versus the cycle number for the CVs shown in (a). Solid lines represent logarithmic curve fitting for oxidation (black) and reduction (red) charge density.

In electrochemical experiments with gold electrodes, the electrochemically active surface area is often estimated by calculating the gold oxide reduction charge.^24^ Therefore, an experiment in a conventional electrochemical cell was conducted on an Au(111) electrode in sulfuric acid. The scan rate was set to 50 mV s^–1^ and the applied potential windows was from 0.98 to 1.73 (V) versus RHE. The current density was calculated by using the geometrical area of the working electrode. Figure 9a shows the CVs measured during the ORCs, with the first cycle shown in blue and the last cycle in red. The first cycles contain a sharp oxidation peak at 1.62 V in agreement with Figure 1b. With increasing cycle number, the sharpness of the main peak decreases and two oxidation peaks at lower potential appear. On the other hand, the shape of the reduction peak remains similar but the decrement of the current density of the peaks is obvious. Figure 9b shows the oxidation and reduction charge densities (μC cm^–2^) as a function of cycle number, obtained by integration of current of the recorded CVs subtracted by the double layer charging current (determined by the current density magnitude at 1 V where neither oxidation nor reduction is taking place). The dots represent the calculated data points and the lines are the result of a logarithmic fitting. The oxidation charge density is in black and the reduction charge is in red. Interestingly, the first cycle shows the highest oxidation and reduction charge density; subsequent ORCs lead to an approximate logarithmic decay with cycle number (fitting results in the SI Table S1).

**Figure 10:**
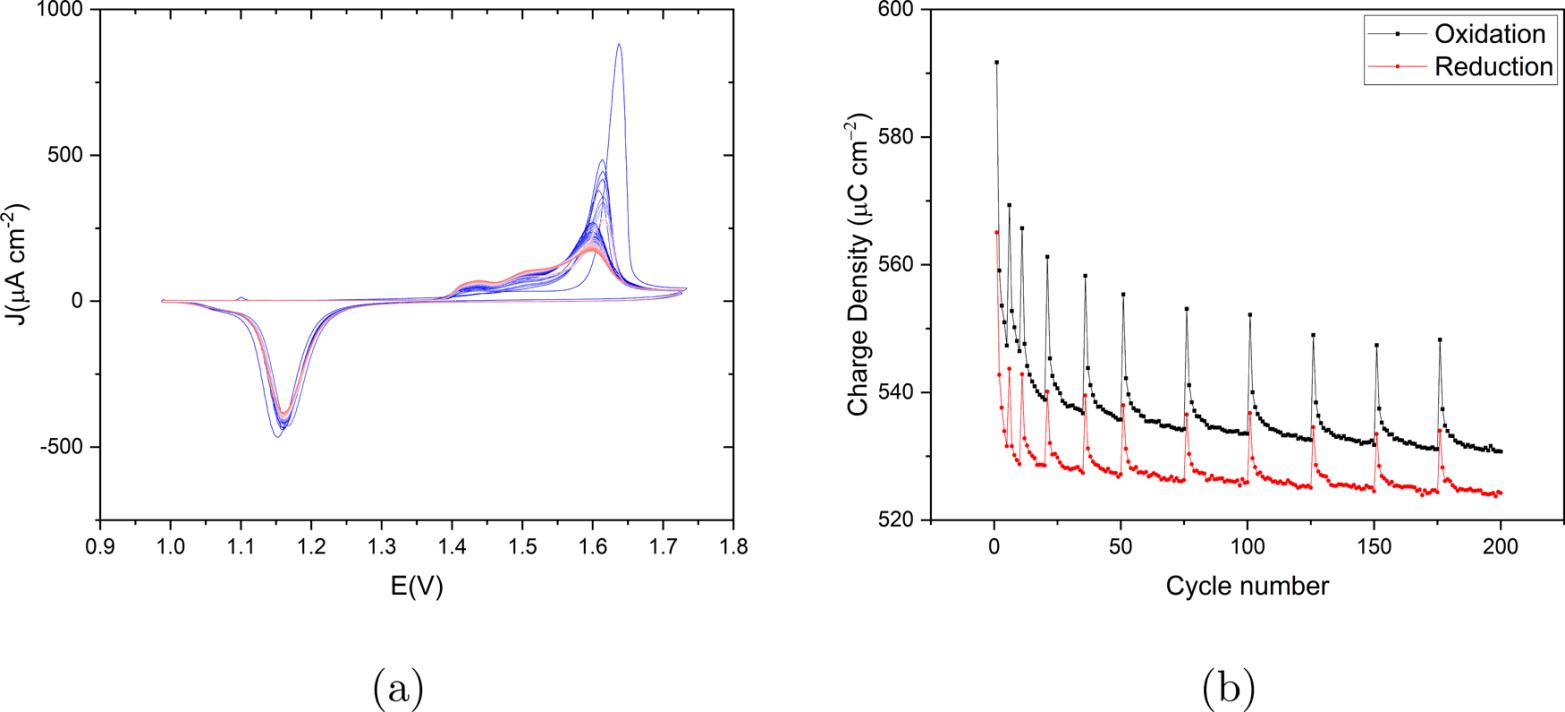
(a) Cyclic voltammogram of the applied 200 ORCs on Au(111) in 0.1 M sulfuric acid with a scan rate of 50 mV s^–1^ versus RHE for the experiment without holding potential in double layer. The color gradient from blue to red corresponds to the progression from the first to the last cycle. (b) Calculated oxidation–reduction charge density (μC cm^–2^) versus the cycle number for the CVs shown in (a).

The same analysis was done on the recorded CVs for the EC-STM experiment without holding the potential in the double layer (the same analysis for the experiment with holding the potential is achievable in the SI). The results in Figure 10a show the CVs, and Figure 10b the calculated oxidation reduction charge density versus cycles number. The oxidation charge is maximum in the first cycle and it reduces gradually. However, a spike in both oxidation and reduction charge density appears just after the STM recording (when the potential is held in the double layer window) which did not appear in the results of the consecutive cycles shown in Figure 9b. The delay is caused by the required time for image recording after the fifth ORC while a constant voltage was applied. Then, the charge decreases again until the 11th cycle after which a new STM image is recorded. Thus, each spike happens in the first cycle after each image recording. Moreover, the magnitude of the spikes decreases with higher cycle numbers. Regardless of the spikes, the overall charge density decreases with increasing number of cycles as in Figure 9b.

It is well-known that extensive electrochemical roughening of the gold surface leads to an increase in oxidation–reduction charge density.^24^ This can be explained by the increase in actual active surface area due to extensive roughening. Considering the calculated roughness value from the two experiments and the oxidation–reduction charge density over cycles, our results reveal an inverse correlation, at least in the roughness regime relevant to our work. This indicates that the atoms on (111) terraces have a higher oxidation charge compared to the defects (different step types around the adatom islands and vacancy islands). Oxidation and reduction charge densities of Au(111) with different terrace widths have been reported in 0.1 M sulfuric acid for the first cycle after annealing.^25^ The reported values for both anodic and cathodic charges showing a decrease for lower terrace width, confirm the difference for oxidation and reduction charges for different sites. This is consistent with the sharp increase observed in the oxidation charge density immediately following the delays, as we noted that the delays reduced the number of smaller islands in the STM images. This increases the proportion of atoms on the (111) terrace relative to the atoms at the defects, and hence the corresponding oxidation/reduction charges. The comparison of our STM images for both experiments with Kolb’s results^3^ reveals a difference in the results at lower ORC numbers. For example, after ten cycles, no adatom islands were reported in their experiments and the entire scanning area was covered by pits, which indicates a higher dissolution rate of gold atoms for the low cycle numbers compared to our experiment. Our results show many monatomic islands (more with more delays) and bilayer islands with some pits between them at the same cycle number. Furthermore, our experiment with delays shows that the duration of the experiment, such as scan rate or image capture time, has a substantial impact on the observed roughening, due to the high surface diffusion rate of the gold atoms, even in the double-layer window. Apart from this difference, the final results after many ORCs match qualitatively in terms of the shape of the islands and the long-range surface roughening.

The formation of nanoislands during ORCs is very similar to what has been observed for a Pt(111) surface,^26^ a kinetic model for which was proposed.^27^ The primary difference is the much higher surface mobility of Au surface adatoms compared to Pt adatoms, including their ability to move over the step sites. Unlike the discussed inverse correlation between oxidation charge and surface roughness for Au(111), the study of the correlation between surface roughness and electrochemical data (hydrogen desorption charge) on Pt(111) showed that after the 31st cycle, every newly formed step site influences both the electrochemical signal and the surface roughness.^26^ In other words, there is an approximately linear correlation between the surface roughness and the hydrogen desorption charge after a certain cycle number. Such a correlation does (unfortunately) not exist for Au(111) roughening.

## Conclusions

In this paper, we performed an in situ EC-STM and electrochemical study of the roughening of an Au(111) electrode in sulfuric acid. Although such (nano)roughening of Au(111) has been studied before,^3^ the atomistic mechanisms and the relation to the observed electrochemical signals have not been elucidated in detail. Specifically, we studied the effect of a delay time by holding the potential in the double layer region, on the roughness development of the surface by calculating the height–height correlation function and compared this with the surface area determination from the oxide formation and reduction charge densities in the CVs. The results suggest that the roughening starts with 2D island formation and is followed by 3D island growth. The extra delays can cause the formation of larger 2D islands (higher correlation length) and a lower roughness amplitude (lower roughening rate per cycle) for both 2D and 3D regimes. By calculating the size of the island, we showed that the number of islands with smaller sizes decreased after the delay, confirming the time effect in Au(111) smoothening caused by the high mobility of gold atoms in the double layer. The oxidation–reduction charge per cycle showed an inverse correlation between the oxide charges and the surface roughness suggesting that the charges at this roughness level are not a good indicator for the actual surface roughness. The results suggest that the oxidation charge per Au site is higher on the (111) terrace compared to other sites. Hence by losing Au(111), the surface charge decreases. The appearance of spikes in oxidation charge after image recording or delays is therefore consistent with a surface smoothening during the potential holding in the double layer.

## Supporting Information Available

Extra experimental information and the general specifications of the designed EC-STM (PDF)

## References

[ref1] Barth J. V.; Brune H.; Ertl G.; Behm R. J. Scanning tunneling microscopy observations on the reconstructed Au(111) surface: Atomic structure, long-range superstructure, rotational domains, and surface defects. Phys. Rev. B 1990, 42, 9307–9318. 10.1103/PhysRevB.42.9307. 9995168

[ref2] Harten U.; Lahee A. M.; Toennies J. P.; Wöll C. Observation of a Soliton Reconstruction of Au(111) by High-Resolution Helium-Atom Diffraction. Phys. Rev. Lett. 1985, 54, 2619–2622. 10.1103/PhysRevLett.54.2619. 10031392

[ref3] Köntje C.; Kolb D. M.; Jerkiewicz G. Roughening and Long-Range Nanopatterning of Au(111) through Potential Cycling in Aqueous Acidic Media. Langmuir 2013, 29, 10272–10278. 10.1021/la4018757. 23855899

[ref4] Schneeweiss M. Oxide formation on Au(111) an in situ STM study. Solid State Ionics 1997, 94, 171–179. 10.1016/S0167-2738(96)00587-5.

[ref5] Conway B. Electrochemical oxide film formation at noble metals as a surface-chemical process. Prog. Surf. Sci. 1995, 49, 331–452. 10.1016/0079-6816(95)00040-6.

[ref6] Kondo T.; Morita J.; Hanaoka K.; Takakusagi S.; Tamura K.; Takahasi M.; Mizuki J.; Uosaki K. Structure of Au(111) and Au(100) Single-Crystal Electrode Surfaces at Various Potentials in Sulfuric Acid Solution Determined by In Situ Surface X-ray Scattering. J. Phys. Chem. C 2007, 111, 13197–13204. 10.1021/jp072601j.

[ref7] Schneeweiss M. A.; Kolb D. M.; Liu D.; Mandler D. Anodic oxidation of Au(111). Can. J. Chem. 1997, 75, 1703–1709. 10.1139/v97-603.

[ref8] Silva F.; Moura C.; Hamelin A. Formation of a monolayer of oxide on gold single crystal face electrodes in sulphamic acid solutions. Electrochim. Acta 1989, 34, 1665–1671. 10.1016/0013-4686(89)85045-5.

[ref9] Diaz-Morales O.; Calle-Vallejo F.; de Munck C.; Koper M. T. M. Electrochemical water splitting by gold: evidence for an oxide decomposition mechanism. Chemical Science 2013, 4, 2334 10.1039/c3sc50301a.

[ref10] Bourkane S.; Gabrielle C.; Huet F.; Keddam M. Investigation of gold oxidation in sulfuric medium—I. Electrochemical impedance techniques. Electrochimica Acta 1993, 38, 1023–1028. 10.1016/0013-4686(93)87022-6.

[ref11] Gao P.; Gosztola D.; Leung L.-W. H.; Weaver M. J. Surface-enhanced Raman scattering at gold electrodes: dependence on electrochemical pretreatment conditions and comparisons with silver. Journal of Electroanalytical Chemistry and Interfacial Electrochemistry 1987, 233, 211–222. 10.1016/0022-0728(87)85017-9.

[ref12] Hanson K. J.; Green M. P. Electrochemical Roughening and Annealing of Au(111) Surfaces in Perchloric and Sulfuric Acid Electrolytes Studied by STM. MRS Online Proc. Library 1991, 237, 323 10.1557/PROC-237-323.

[ref13] Wieckowski A. Interfacial Electrochemistry; Routledge: 2017; pp 151–173.

[ref14] Hermann J. M.; Abdelrahman A.; Jacob T.; Kibler L. A. Potential-dependent reconstruction kinetics probed by HER on Au(111) electrodes. Electrochim. Acta 2020, 347, 136287 10.1016/j.electacta.2020.136287.

[ref15] Yang S.; Hetterscheid D. G. H. Redefinition of the Active Species and the Mechanism of the Oxygen Evolution Reaction on Gold Oxide. ACS Catal. 2020, 10, 12582–12589. 10.1021/acscatal.0c03548.

[ref16] Palasantzas G.; Krim J. Effect of the form of the height-height correlation function on diffuse x-ray scattering from a self-affine surface. Phys. Rev. B 1993, 48, 2873–2877. 10.1103/PhysRevB.48.2873. 10008702

[ref17] Krim J.; Indekeu J. O. Roughness exponents: A paradox resolved. Phys. Rev. E 1993, 48, 1576–1578. 10.1103/PhysRevE.48.1576. 9960753

[ref18] Le Gal A.; Klüppel M. Investigation and modelling of rubber stationary friction on rough surfaces. J. Phys.: Condens. Matter 2008, 20, 015007 10.1088/0953-8984/20/01/015007.

[ref19] Li P.; Ding F. Origin of the herringbone reconstruction of Au(111) surface at the atomic scale. Sci. Adv. 2022, 8, eabq2900 10.1126/sciadv.abq2900. 36197981 PMC9534511

[ref20] Giesen M.; Schulze Icking-Konert G.; Ibach H. Fast Decay of Adatom Islands and Mounds on Cu(111): A New Effective Channel for Interlayer Mass Transport. Phys. Rev. Lett. 1998, 80, 552–555. 10.1103/PhysRevLett.80.552.

[ref21] Giesen M.; Schulze Icking-Konert G.; Ibach H. Interlayer Mass Transport and Quantum Confinement of Electronic States. Phys. Rev. Lett. 1999, 82, 3101–3104. 10.1103/PhysRevLett.82.3101.

[ref22] Giesen M.; Ibach H. On the mechanism of rapid mound decay. Surf. Sci. 2000, 464, L697–L702. 10.1016/S0039-6028(00)00693-2.

[ref23] Nečas D.; Klapetek P. Gwyddion: an open-source software for SPM data analysis. Open Physics 2012, 10, 181–188. 10.2478/s11534-011-0096-2.

[ref24] Wang W.; Huang Y.-F.; Liu D.-Y.; Wang F.-F.; Tian Z.-Q.; Zhan D. Electrochemically roughened gold microelectrode for surface-enhanced Raman spectroscopy. J. Electroanal. Chem. 2016, 779, 126–130. 10.1016/j.jelechem.2016.04.008.

[ref25] Zhumaev U.; Rudnev A. V.; Li J.-F.; Kuzume A.; Vu T.-H.; Wandlowski T. Electro-oxidation of Au(111) in contact with aqueous electrolytes: New insight from in situ vibration spectroscopy. Electrochim. Acta 2013, 112, 853–863. 10.1016/j.electacta.2013.02.105.

[ref26] Jacobse L.; Huang Y.-F.; Koper M. T. M.; Rost M. J. Correlation of surface site formation to nanoisland growth in the electrochemical roughening of Pt(111). Nat. Mater. 2018, 17, 277–282. 10.1038/s41563-017-0015-z. 29434306

[ref27] Rost M. J.; Jacobse L.; Koper M. T. M. The dualism between adatom- and vacancy-based single crystal growth models. Nat. Commun. 2019, 10, 5233 10.1038/s41467-019-13188-0. 31748552 PMC6868172

