# Supplementary material for: In Situ STM Study of Roughening of Au(111) Single-Crystal Electrode in Sulfuric Acid Solution during Oxidation–Reduction Cycles
Source: J Phys Chem C Nanomater Interfaces. 2024 Oct 17;128(44):19024–34. doi: 10.1021/acs.jpcc.4c06362 (PMC11552071; doi:10.1021/acs.jpcc.4c06362)
Supplement: Supplementary file 1 — jp4c06362_si_001.pdf [file jp4c06362_si_001.pdf]

Supporting Information to: In situ STM Study of Roughening of Au(111) Single-Crystal Electrode in Sulfuric Acid Solution during Oxidation-Reduction Cycles

Saeid Behjati* and Marc T.M. Koper*

Leiden Institute of Chemistry, Leiden University, PO Box 9502, 2300 RA Leiden, the Netherlands

E-mail: s.behjati@lic.leidenuniv.nl; m.koper@lic.leidenuniv.nl

Lifting the reconstruction for the experiment with holding the potential

The EC-STM image in Figure S1a was initially recorded at 0 V just after the thermal annealing. The stripe reconstruction is visible, indicating that the sample is in good condition to start the experiment. There is a screw dislocation at the bottom-left part of the image which is a result of a defect in the crystal.[1] Subsequently, a voltage sweep from 0 to 0.2 V was applied, and Figure S1b was recorded at 0.2 V. The image shows some defects/islands forming almost at the center of the imaged region. At this potential, we do not expect the lifting of the reconstruction since the electrode potential is lower than the potential of zero charge. It is possible to see the stripe reconstruction near the formed island which proves that the island formation cannot be due to the lifting of the reconstruction. After the potential

was swept from 0.2 to 0.4 V, Figure S1c was recorded at 0.4 V, depicting the two islands as a defect at the center. After sweeping the potential to 0.6 V, Figure S1d was recorded, showing the generation of the small islands in the defect area. With a further sweep to 0.7 V, Figure S1e was recorded, indicating some major changes in the step lines. It can be seen that the reconstruction lines are not as impacted as the pristine surface and they are not entirely parallel anymore. At 0.8 V, Figure S1f was recorded, and lifting the reconstruction led to the formation of small islands all over the terraces. At 0.88 V and 0.98 V, Figure S1g and 1h were recorded, respectively. By comparing Figure S 1f and 1h, one can conclude that the island size increased at the expense of the smaller islands on the large terraces.

HHCF result for the instant frames

Figure S2a shows the HHCF for the instant frames in the experiment with holding potential in the double layer. As expected, increasing the cycle number increases the roughness magnitude and the correlation length shifts toward a higher radius. The roughness amplitude and correlation length versus cycle number for instant frames are shown in Figure S2b revealing the linear behavior for roughness amplitude and a non-linear behavior for correlation length. At the initial stage of roughening, the islands grow in the 2D regime (corresponding to the correlation length), and as soon as they reach the size of ca. 20 nm, their lateral size changes more slowly and they tend to grow in the 3D regime.

Curve fitting for OR charges

Equation1 is used to achieve the best curve fitting and the acquired coefficients are listed in Table 1. The offset value (a) for reduction charge density is 7.8 (µC cm^{-2}) less than oxidation charge density and it can be related to the dissolution of gold in either anodic sweep[2] or reduction of gold oxide during cathodic sweep,[3,4] or in both directions.[5] The reported charge density difference per cycle in 0.5 M sulfuric acid is 4.2 (µC cm^{-2}) of which 3.7 (µC cm^{-2})

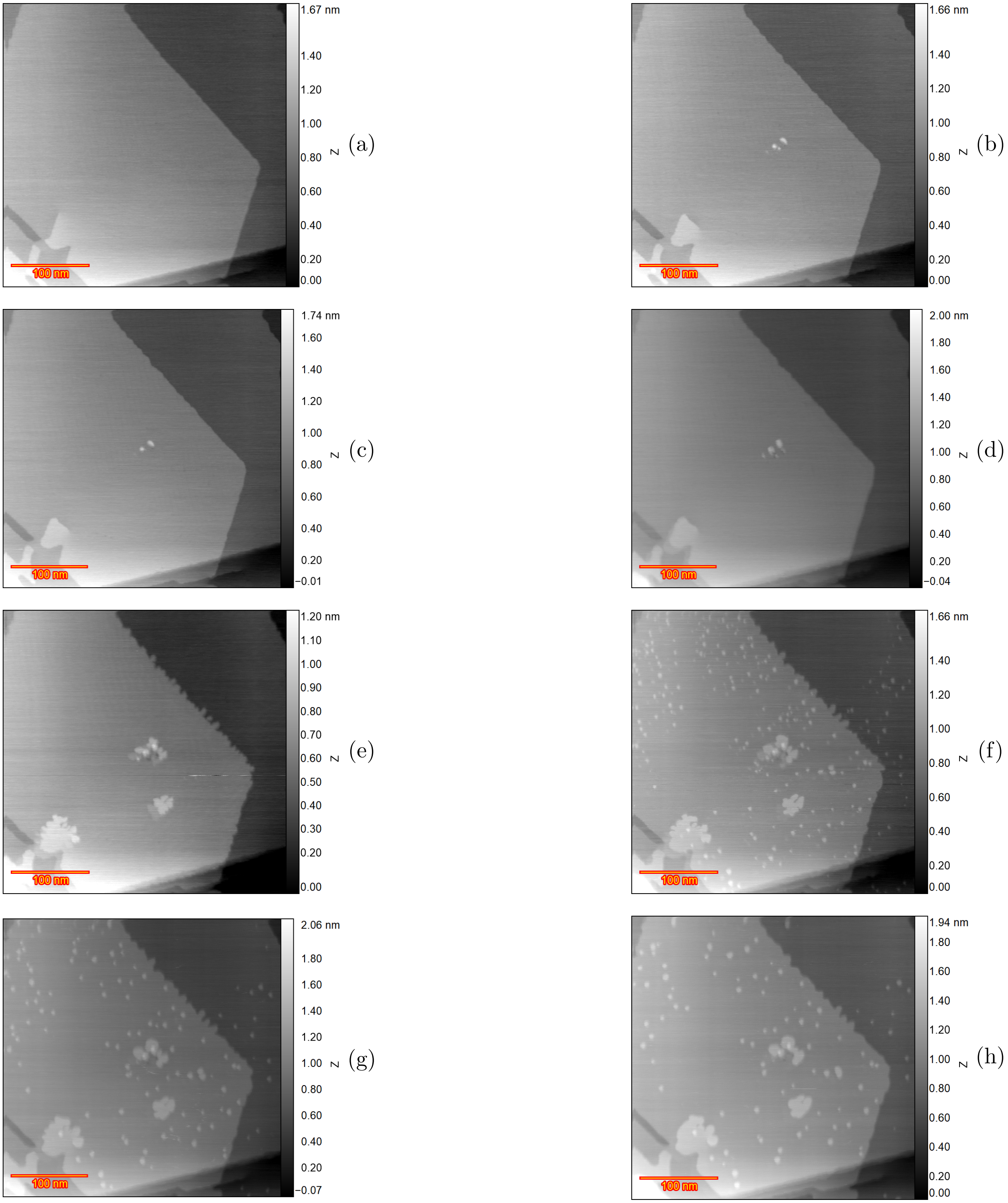

Figure S 1: Au(111) in 0.1M sulfuric acid with the image size of 350×350 nm at different potentials a) 0, b) 0.2, c) 0.4, d) 0.6, e) 0.7, f) 0.8, g) 0.88, and h) 0.98 V versus RHE.

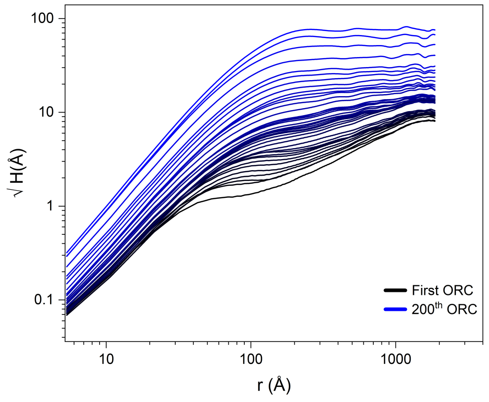

(a)

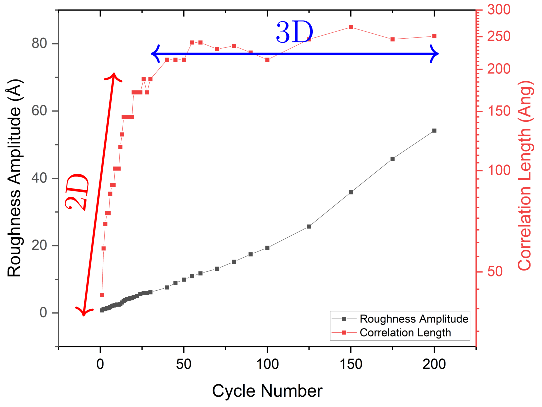

(b)

Figure S 2: a) Height-height correlation function versus distance r for Au(111) in 0.1M sulfuric acid as a function of the number of oxidation-reduction cycles (ORCs) for the experiment with holding potential in the double layer for the instant frames. b) Extracted roughness amplitude and correlation length versus cycle number from the HHCF results. The arrows indicate the 2D and 3D island growth regimes.

is caused by anodic sweep and 0.5 (µC cm^{-2}) by cathodic sweep.[5] The difference in offset value (a) for oxidation and reduction in our study is almost double the reported amount.[5] To have a better understanding of this happening, this difference versus cycle number is plotted in Figure S3. This value is the highest for the first cycle and it decreases rapidly to 7 (µC cm^{-2}) and it continues to decrease over cycles. At the last cycle, it reaches 4.83 (µC cm^{-2}). This also can explain the minor difference in the logarithm coefficient (b) for oxidation and reduction in Table S1. With the reported coefficients and the fact that there is a linear correlation between the cycle number and the surface roughness, one can relate the oxidation or reduction charge density to the roughness value.

$$y = a - b \times ln(x + c) \tag{1}$$

Table S 1: The calculated coefficients for curve-fitting on the oxidation-reduction charge density shown in Figure 9b by using Equation1.

**Reduction Coefficients**	**Oxidation Coefficients**
a = 561.47713 ± 0.39641	a = 569.28624 ± 0.41304
b = 5.29827 ± 0.08969	b = 5.76885 ± 0.09353
c = -0.9771 ± 0.00608	c = -0.99642 ± 9.70464E-4

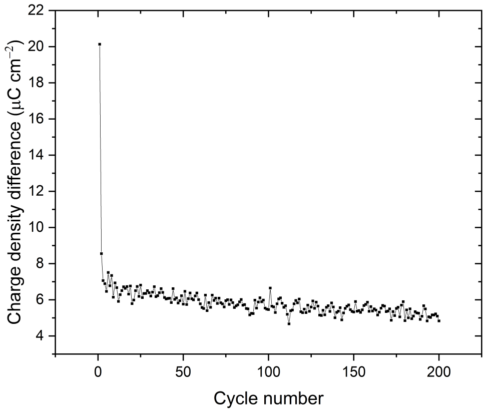

Figure S 3: Calculated the difference in oxidation-reduction charge density (µC cm^{-2}) in cyclic voltammogram of the consecutively applied 200 ORCs on Au(111) in 0.1M H2SO4 with a scan rate of 50 mV s^{-1} versus RHE.

Oxidation-Reduction charge density with holding the potential

Oxidation-Reduction charge density for the experiment with holding potential in the double layer is shown in Figure S4a. In this experiment, more delays were applied, and as a result, different CVs and oxidation-reduction charge densities are expected. The amplitude of the main oxidation peak for the first and second cycles is not as noticeable as for the aforementioned results due to the applied delay. This behavior can also be seen in Figure S4b as the charge density does not drop as fast as in other experiments. For the first twenty cycles no spike can be seen in the charge density since after each cycle the delay has been applied. However, after the twentieth cycle, the spikes appeared on the cycles subsequent to the delays. Regardless of the spikes, the general trend is an approximately logarithmic

decay of the charge density over cycles.

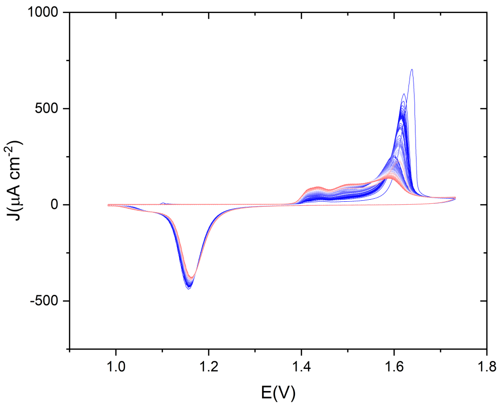

(a)

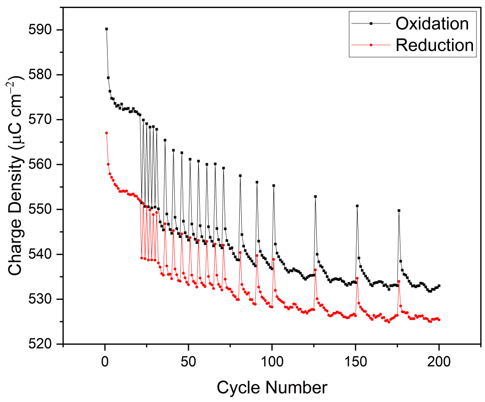

(b)

Figure S 4: a) Cyclic voltammogram of the applied 200 ORCs on Au(111) in 0.1M sulfuric acid with a scan rate of 50 mVs^{-1} versus RHE for the experiment with holding potential in double layer. The color gradient from blue to red corresponds to the progression from the first to the last cycle. b) Calculated oxidation-reduction charge density (µC cm^{-2}) versus the cycle number for the CVs shown in (a).

EC-STM setup

This section provides information about the recently designed EC-STM instrument at the Leiden Institute of Chemistry (LIC).

There are many different mechanical designs for the SPM which were introduced thoroughly in the literature.[6] EC-STM has some specific challenges and these should be considered during the design steps. The main considerations are as follows:

- The sample should always placed horizontally and the tip should land on the sample from the top since the surface should be in contact with the electrolyte.
- The electrolytes can be very corrosive and damaging to the mechanical parts. Protective properties are essential.

- The electrolyte should be in contact with as few parts as possible since the cleanliness of the entire electrochemical cell is crucial and those parts should be resistive against the electrolytes and should not contribute to the electrochemical reactions inside the cell.
- Easy and fast assembling steps for the experiments because the complexity of these steps can lead to introducing extra contamination to the sample surface and the electrochemical cell.

The best fit for these requirements is the single-tube scanning tunneling microscope design. In this design,[7] the single tube scanner head is placed on the top of the sample and secured with springs. The tip is hanging on the single tube translator (scanner head) and it can land vertically on the sample. The sample and the entire electrochemical setup can be installed on the base plate. By removing the single tube transducer, the operator has enough room to assemble the sample and EC cell quickly. Three fine screws help the coarse approach steps and the fine approach step can be done by the accurate motors.

The cross-section of the design in the Y-Z plane crossing the center of the sample is shown in Figure S5. The EC cell is in yellow and the insulator plate is in blue. The base plate is located at the bottom and the tip (in green) is hanging from the tip holder. The selected material for the tip holder, the EC cell, and the insulator plate is PEEK since PEEK is a very good electrochemically resilient material for basic and acidic electrolytes and it is suitable for a large spectrum of electrochemical experiments. PEEK is also resilient against the convectional cleaning procedure (soaking in potassium permanganate, then diluted piranha solution, and several times boiling in milli-Q water). The O-ring in black is located above the sample and underneath the EC cell to avoid electrolyte leakage and the inlet and outlet are located on the left and right side of the EC cell. Peristaltic pumps allow the pumping of the fresh and degassed electrolyte into the cell and the draining of the used electrolyte from the cell.

The electrochemical cell is surrounded by a chamber with constant Argon flow during the

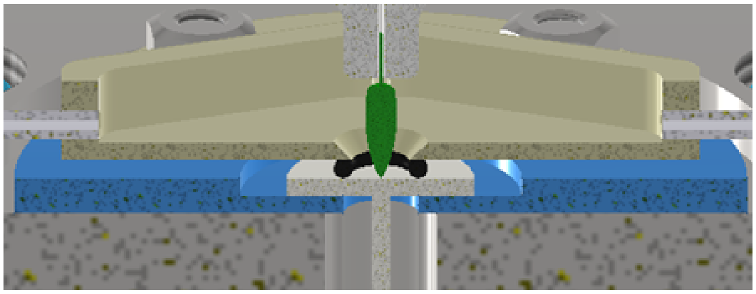

Figure S 5: Cross section of the design in Y-Z plane. The EC cell is in yellow and the insulator plate is in blue. The base plate is located at the bottom and the tip (in green) is hanging from the tip holder. The O-ring in black is located above the sample and underneath the EC cell to avoid electrolyte leakage and the inlet and outlet are located on the left and right side of the EC cell.

runtime to reduce the chance of introducing/dissolving unwanted gasses into the electrolyte. Like other SPM techniques, EC-STM needs some mechanical noise reduction/insulator system. A passive vibration isolation system housed the instrument and led to satisfactory results in a location not exposed to extensive mechanical vibrations during the runtime. Acoustic noise also needs to be avoided for high-quality results.

Electrical design

Many electrical modules with specific characteristics are essential in order to have a functional EC-STM like potentiostat, controller, analog module, and preamplifier.

Figure S 6 shows the schematics of the designed potentiostat. On the left-hand side, four inputs are shown. The two top inputs will set the electrochemical voltage and can accept two AC and DC signals. The DC input is suitable for low-speed changing waveform, like a conventional triangular waveform for a cyclic voltammogram. The AC input can be used for other fast-changing signals and it helps to deploy ACV techniques. The two bottom inputs can adjust the working electrode potential, which in a conventional potentiostat is either grounded or remains floating. With this configuration, the WE potential can be adjusted precisely by a DC voltage or with an AC potential to apply modulation techniques to the tunneling bias. The working electrode is connected to a transimpedance amplifier to convert

the current on the WE into a readable voltage which can be read at WE I_{Out} connector. There are seven different current ranges available and can be changed digitally from $1\mu A$ to $200mA$. With $1\mu A$ as the current range setting, nano ampere currents can be read. These gain settings can also influence the bandwidth and phase margin of the potentiostat. As a rule of thumb, higher transimpedance gains will cause a reduction in the bandwidth. Another operational amplifier with a voltage follower configuration is connected to the WE to read the actual voltage. This is important because the bandwidth limitations and wrong gain settings can lead to a strong deviation from the asked WE potential. To improve the reliability and precision of the potentiostat the WE potential is always read directly by that circuit. This voltage is detectable at WE U_{Out} connector. The reference electrode is directly connected to the non-inverting input of the operational amplifier with a voltage follower configuration. This makes reading of the Ref potential possible with great precision and the output is connected to Ref U_{Out}. The final part is the counter electrode driver module which reads the EC potential setpoint, the reference electrode voltage, and the actual working electrode potential to adjust the counter electrode voltage so that the voltage difference between the working electrode and the reference electrode matches with the asked EC potential. The bandwidth of this module is adjustable with four digitally controlled channels. The main specification of the designed potentiostat is as follows:

- Tunable bandwidth for CE.
- Tunable bandwidth and gain for WE.
- Maximum 5 pA RE input current (very low ohmic error).
- Second-order low-pass active filter on inputs.
- AC and DC inputs for each channel (AC for modulation and DC for voltage sweep).
- 200 mA continues output current.
- Control of CMOS switches with 10 ns resolution.

- Everything is controlled by an FPGA (no selector or knob) and the instrument can be run remotely.

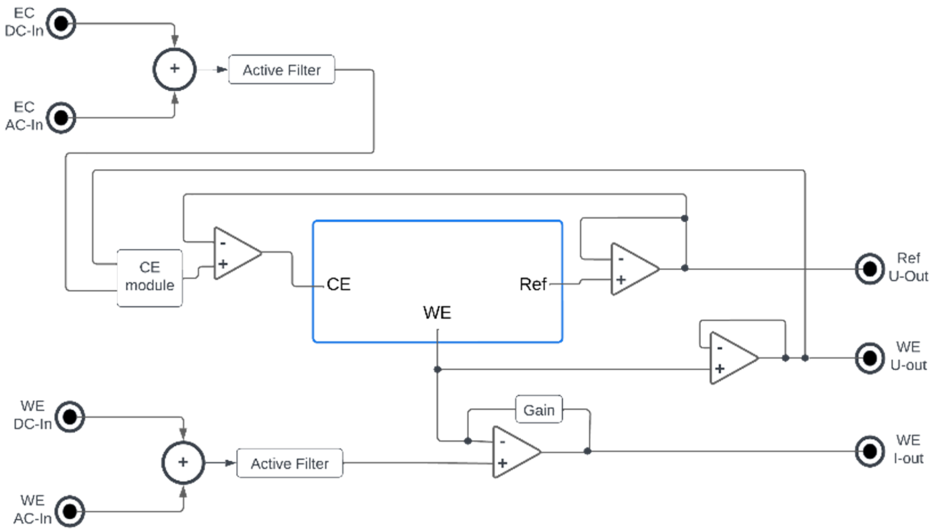

Figure S 6: Potentiostat simple schematics with the cell containing the electrodes in the middle. On the left, there are four input channels, and on the right, three outputs are shown.

By using a National Instrument DAQ card with the potentiostat all the waveforms can be generated and passed to the potentiostat and all the output voltages from the potentiostat can be read by the DAQ card. A home-built software is developed to run electrochemical experiments with different techniques (e.g. CA, and CV) then process, plot, and save the data. Due to the AC inputs on potentiostat, more complicated measurement techniques can be developed and deployed. As the controller, MK3 from Soft dB company has been used. The low price and open-source software made this controller the best option for this version of the EC-STM. The acquired results are satisfactory but more sophisticated electronics can improve the performance of the instrument if needed. There are eight inputs and eight outputs with 16 bits of precision on this controller. The channels can be assigned to different input and output signals which give the operator the ability to conduct many different experiments and techniques. The analog module was designed and manufactured to

take care of all the analog operations on the input and output signals. This module has two main parts and each part has its sub-modules. The first part is called “XYZ modules” and is for all the analog operations on control signals for the piezoelectric in the X, Y, and Z axis. The second part is related to the generation of control signals and some necessary arithmetic operations on the tunneling current signal. These two parts will be discussed in more detail in the following. The controlling signals for X, Y, and Z can be made in the main controller but more essential operations are needed. First, since the controller is capable of generating signals from -10 to + 10 volts with 16 bits precision, in some experiments it is required to reduce the voltage span to increase the precision further. One application for this operation is precision improvement for small scan areas and atomically flat surfaces. In this case, the instrument should have very high resolution in the X, Y, and Z directions. This can be achieved by division of the signals by a certain coefficient. Second, in the case that one wants to zoom in on a small area (compared to the total travel range of the piezoelectric tube), it is important to separate the scanning signal and the offset signal. By applying this, one can record high-precision pictures on a small area while having access to the whole travel range of the piezoelectric tube. The offset signal is also useful for compensating the thermal drifts in all three directions during long experiments. Moreover, in some cases, one needs to apply some modulation in different directions with a certain frequency. So, external modulation signals need to be added to the offset and scanning signals. Third, the out-of-range potential of the piezo is damaging and needs to be avoided. Since commercial high-voltage amplifiers usually have a fixed gain value, it is wise to have good control of the output voltage levels. Fourth, a high order low pass filter can reduce the voltage noise level on the output since the output of this module will be connected to the high voltage amplifier, the voltage noise will be magnified by the gain value of the high voltage amplifier and this magnified noise can reduce the precision of the tip location in three axes. Additionally, the low pass filter can avoid the first fundamental resonance frequency of the piezoelectric tube to avoid unwanted oscillation during the experiments by cutting the sharp edge of the triangular waveform.

The general specifications of the module are as follows:

- Differential or single-ended signals on all of the inputs.
- Ultra-low noise linear voltage regulators on the power lines.
- Ultra-low noise and drift +10 and -10 volts reference for the offsets.
- Adjustable minimum and maximum voltage for the low pass filter to limit the output voltage (to avoid damaging the piezo).
- 7th and 8th order low pass filter to reduce the noise and cut the sharp edges of the scanning waveform. The cutoff frequency and the topology of the low-pass filter can be designed and implemented as per the project requirements.
- The internal offsets and the attenuation coefficient are controlled by a computer in 256 steps (LabVIEW or other languages).
- Ability to apply both internal and external offset signals for all directions.
- The maximum bandwidth of the attenuators is 500kHz. If higher bandwidth is required, a regular potentiometer can be installed.
- Input for AC signals in case of modulation in different directions.

Since the tunneling current can flow in two directions depending on the polarity of the tunneling bias, both positive and negative signals are possible at the output of the pre-amplifier. The opposite directions for negative and positive signals can cause trouble for the feedback loop. An absolute amplifier is included in the analog module to rectify that. Moreover, the tunneling current magnitude is an exponential function of the tip-sample distance, having a logarithmic amplifier is a must. The logarithmic amp can perform logarithm operations over 8 decades (very wide dynamic range). Finally, a PID controller with a tunable active low pass filter is included to be used as an analog feedback loop.

A special pre-amplifier module with two working modes was designed. The first mode acts as a transimpedance amplifier with a gain of 10^9 vA^{-1} with an output voltage range of ± 10 v ends up accepting ± 10 nA at the input. The bandwidth of this TIA is set to 30 kHz since it is sufficient for normal experiments but the design bandwidth is 100 kHz. Thus, depending on the experiment requirements, the bandwidth can be increased. The second mode acts as a voltage follower and this helps to put the tip in open circuit configuration and reading the tip potential is feasible. The bandwidth for this mode is 1 MHz and the maximum required current at the input is 2 pA. Switching between the modes can be applied remotely and this process does not make any mechanical vibration/noise. Thus, switching is possible during the EC-STM experiments. For the conventional EC-STM experiments, only the first mode is required.

References

(1) Li, S.; Wang, G. *Introduction to Micromechanics and Nanomechanics*; WORLD SCIENTIFIC, 2018; pp 341–411.

(2) Cherevko, S.; Topalov, A. A.; Zeradjanin, A. R.; Katsounaros, I.; Mayrhofer, K. J. J. Gold dissolution: towards understanding of noble metal corrosion. *RSC Advances* **2013**, *3*, 16516.

(3) Cadle, S. H.; Bruckenstein, S. Ring-disk electrode study of the anodic behavior of gold in 0.2m Sulfuric acid. *Analytical Chemistry* **1974**, *46*, 16–20.

(4) Vesztergom, S.; Ujvári, M.; Láng, G. RRDE experiments with potential scans at the ring and disk electrodes. *Electrochemistry Communications* **2011**, *13*, 378–381.

(5) Shrestha, B. R.; Nishikata, A.; Tsuru, T. Application of channel flow double electrode to the study on gold dissolution during potential cycling in sulfuric acid solution. *Journal of Electroanalytical Chemistry* **2012**, *665*, 33–37.

(6) Chen, C. J. *Introduction to Scanning Tunneling Microscopy*, 3rd ed.; Oxford University PressOxford, 2021.

(7) Drake, B.; Sonnenfeld, R.; Schneir, J.; Hansma, P. Scanning tunneling microscopy of processes at liquid-solid interfaces. *Surface Science* **1987**, *181*, 92–97.
